# Complete Genome Sequence of Variant Porcine Epidemic Diarrhea Virus Strain ZJ/ZX2018-C10, Isolated in Zhejiang, China, in 2018

**DOI:** 10.1128/MRA.00048-19

**Published:** 2019-03-21

**Authors:** Fei Su, Bin Yu, Junxing Li, Lihua Xu, Xiufang Yuan

**Affiliations:** a Zhejiang Academy of Agricultural Sciences, Institute of Animal Husbandry and Veterinary Science, Hangzhou, Zhejiang, China; Broad Institute of MIT and Harvard University

## Abstract

We report here the complete genome sequence of porcine epidemic diarrhea virus (PEDV) strain ZJ/ZX2018-C10, isolated from infected piglets in Zhejiang Province, China. The genome sequence was highly similar to AH2012, a highly virulent Chinese PEDV strain. It will help in understanding the molecular and evolutionary characteristics of PEDV in China.

## Abstract (precis)

We report here the complete genome sequence of porcine epidemic diarrhea virus (PEDV) strain ZJ/ZX2018-C10, isolated from infected piglets in Zhejiang Province, China. The genome sequence was highly similar to AH2012, a highly virulent Chinese PEDV strain.

## ANNOUNCEMENT

Porcine epidemic diarrhea (PED) is an acute and highly contagious enteric disease caused by porcine epidemic diarrhea virus (PEDV), which leads to mild to severe watery diarrhea, vomiting, and dehydration in pigs of all ages and a high mortality rate in neonatal piglets (1, 2). PEDV is an enveloped single-stranded RNA virus that belongs to the genus *Alphacoronavirus* of the family *Coronaviridae* (3, 4). Although PED has been reported in China since the 1980s, it was sporadic and regional before 2010 (1). In 2010, a large-scale outbreak of PED with high morbidity and mortality swept across China, leading to enormous economic losses (5–7).

In February 2018, a stool sample obtained from a naturally infected piglet from Zhejiang Province in China was processed and determined to be positive for PEDV by reverse transcription-PCR (RT-PCR) with primers targeting the N gene. A PEDV strain named ZJ/ZX2018-C10 was isolated using Vero cell cultures as previously described (8). Viral RNA was extracted from the cell supernatant using the Macherey-Nagel viral RNA purification kit (Macherey-Nagel GmbH & Co. KG, Germany) according to the manufacturer’s instructions. To determine the complete genomic sequence of the PEDV isolate of strain ZJ/ZX2018-C10, the extreme 5′ and 3′ termini were acquired by rapid amplification of cDNA ends (RACE, Clontech, Japan). Subsequently, a total of 13 overlapping fragments were amplified by RT-PCR using PrimeSTAR hot-start (HS) DNA polymerase (TaKaRa, China) with primers (Table 1) based upon the sequences of PEDV strain CV777 (GenBank accession number AF353511). The PCR products were analyzed and confirmed on 1% agarose gel and cleaned with a DNA fragment purification kit (TaKaRa, China). Purified PCR products were cloned into the pMD19-T vector and sequenced in both directions with the Sanger sequencing method (Biosune Biotechnology Shanghai Co., China) on the ABI 3730xl DNA analyzer platform (Life Technologies). For each amplification, three to five individual clones were sequenced to determine the consensus sequence of any given genomic region. Sequence contigs with the consensus sequence were assembled into the full-length genome for the PEDV isolate of strain ZJ/ZX2018-C10 using Lasergene version 7.10 (DNAStar, Inc., USA).

**TABLE 1:** Primers used for amplifications of the PEDV genomic fragments

Primer name	Sequence (5′ to 3′)	Position^*a*^	Product size (no. of nucleotides)
1F	GCGTTCCGTCGCCTTCTACA	190–209	2,562
1R	CAGGAATCTGGAAGACACTTGCA	2751–2729	
2F	GTATTATGCCACCAGTGTCCCA	2663–2684	2,295
2R	CAGTTGCCAGCAGGCACTGT	4957–4938	
3F	ACCAGCGGTGCATTGCTTGA	4887–4906	2,589
3R	CAATGTGCTCTTGCAATCCTGCA	7475–7453	
4F	CTGTTAAGTTAGTGGACTCAGCGT	7327–7350	2,549
4R	ACTAGCGCCTTCAACTTGCA	9875–9856	
5F	GCGCTTGTGGTTCACCTGGT	9712–9731	2,547
5R	GGATCCACAGCGAAAGCGCA	12259–12240	
6F	ACGCTTGCAGGCTGGTAAACA	12182–12202	2,281
6R	TGGGCAGTGCTCTATCGCACT	14462–14442	
7F	ATACTAGGGGCGCTTCGGTT	14322–14341	2,459
7R	GTCAGGGTGCACAGGAATGAA	16780–16760	
8F	GTATGTGTGCCCTTAAGCCTGAT	16662–16684	2,341
8R	GTAAGTGGACGTTCGGCTTCATA	19002–18980	
9F	TGTATGCCAAGCGTAAGGTAGGAC	18888–18911	1,816
9R	ATCTTGTGGTAGGCTGAGTGTTGG	20703–20680	
10F	GAAGAATGGTAAGTTGCTAGTGCGTA	20563–20588	2,130
10R	GGCTAACAACTGTCCAGAATCAGATG	22683–22658	
11F	AAAGGTGAGTTGATTACTGGCACG	22498–22521	2,164
11R	CTAGTAATGACACAACAAAGATGAGAAC	24664–24637	
12F	GTGTACGATCCTGCAAGTGGC	23272–23292	2,439
12R	TCACCTCATCAACGGGAATAGA	25715–25694	
13F	TCGTCCAATTGGTTAATCTGTGC	25535–25557	2,306
13R	TACCGTTGTGTGCAAGACCAA	27840–27820	
5′ RACE	TCCACTAGCGGCGGCCTCAGAATA	410–387	185
3′ RACE	TCAACGAGATCTTCGATACAGGAA	27671–27694	193

a Positions correspond to strain CV777 (GenBank accession number AF353511).

The complete genome sequence of strain ZJ/ZX2018-C10 is 28,035 nucleotide (nt) bases long, excluding the poly(A) tail, with a G+C content of 41.72%. The open reading frames for ZJ/ZX2018-C10 were predicted using Genome Annotation Transfer Utility with PEDV strain CV777 as the reference genome. The genomic organization of the virus is in the following order: 5′ untranslated region (UTR), nucleotide 1 to 292; replicase polyprotein, nucleotide 293 to 12616 for 1a and nucleotide 12616 to 20637 for 1b; spike (S) protein, nucleotide 20634 to 24791; open reading frame 3 (ORF3), nucleotide 24791 to 25465; envelope (E) protein, nucleotide 25446 to 25676; membrane (M) protein, nucleotide 25684 to 26364; nucleocapsid (N), nucleotide 26376 to 27701; and 3′ UTR, nucleotide 27702 to 28035. The complete genome sequence of ZJ/ZX2018-C10 shares 98.4%, 97.1%, and 96.3% nucleotide sequence identities with strain AH2012 (GenBank accession number KC210145), strain virulent DR13 (GenBank accession number JQ023161), and vaccine strain CV777 (GenBank accession number KT323979), respectively. Interestingly, strain ZJ/ZX2018-C10 possesses one unique deletion site compared to strain AH2012 (nucleotide 24225 to 24227) in the S gene, resulting in a single-amino-acid deletion (^1198^T). Of note, the deletion site is in the S2 domain of the S protein. These findings suggest that ZJ/ZX2018-C10 is a novel PEDV variant. The genome information of strain ZJ/ZX2018-C10 will promote a better understanding of the evolutionary characteristics and molecular epidemiology of PEDV in China.

### Data availability.

The genome sequence of PEDV strain ZJ/ZX2018-C10 has been deposited in GenBank under the accession number MK250953.
